# Supplementary material for: Patterns of Intron Gain and Loss in Fungi
Source: PLoS Biol. 2004 Nov 30;2(12):e422. doi: 10.1371/journal.pbio.0020422 (PMC532390; doi:10.1371/journal.pbio.0020422)
Supplement: Table S1 — Also available at http://genes.mit.edu/NielsenEtAl/. (4.3 MB ZIP). [file pbio.0020422.st001.zip › NielsenEtAl/html/503.html]

AN7307.1.NCU09737.1.MG04128.1.FG04255.1


```
 CLUSTAL W (1.82) Multiple Sequence Alignments - Introns Inserted


Sequence 1: NCU09737.1	552 aa
Sequence 2: MG04128.1	539 aa
Sequence 3: FG04255.1	543 aa
Sequence 4: AN7307.1	523 aa
Alignment Length: 569 aa
Number Identitical Residues: 241 aa
Alignment Score (without introns) 12104


MG04128.1 	-MGPTKFLFLLP------LLAHLAEANAG--DSAQVPLHG-FEHAACPDYASYSKYKH2P
NCU09737.1	MTRATSWLSLLLPWSVPLLRPAVVEASELKWDMDQVPIKGKFDETACPDYASYATYPH2R
FG04255.1 	-----MTLCLLAS----SLLALSATLASASPDDEQIPLPD--YRRICPDYTRYAAFPH2R
AN7307.1  	MTLPALVL-ALS------LLSANAREAAALRDEPTI------LRRACPDYLSYSTAP-~-
          	   .:  *  *       * .  .       *   :       .  ****  *:      

MG04128.1 	PFSDGPLKLPYQR~PDPRCRTFNNDAIEKVIKDVTSRMKDPDMARLFENAFPSTTDTTVK
NCU09737.1	PYSEGPLSLPFQR~PHVRCRTFQSDAIEKVIKDVTSRMKNPDLARLFENTFPSTTDTTVK
FG04255.1 	PFSTGPKALPYQR~PAKQCRTFQSDVIEKVIEDVTSRMKDPDLARLFENAFPSTTDTTVK
AN7307.1  	------------Q2PAIECRTFNSSAVEQVIEDVTSRMIDKDLAQLFRNAFPNTLDTTIR
          	            : *  .****:...:*:**:****** : *:*:**.*:**.* ***::

MG04128.1 	FHTDG-------KTKHRSKPHDEDDDFFGFGTDSKGAWEGPQSFIITGDIIAEWLRDSTN
NCU09737.1	FHTNGKDGKEKRKTTTVTDSPVDDDPWTSTYEEDPGKWEGPQSFIITGDILAEWLRDSTN
FG04255.1 	FHTKG-------KTDTG---FFRMGSFGGFH--DEGAWEGPQSFIITGDIIAEWLRDSTN
AN7307.1  	WHIDG----------------FTTAIRQSKSKKQNEQWTGPQTFVVTGDINAEWLRDSTN
          	:* .*                       .   ..   * ***:*::**** *********

MG04128.1 	QLAPYQPLAQKNQAVRTLILGAINTQAEYIIQSPYCNAFQPPPIANLPISHNGQDDTVHP
NCU09737.1	QLKPYQKLASKDKAIFDLILGAINTQSEYVIESPYCNAFQPPPHSGLPVTLNGQDDTVHP
FG04255.1 	QLRPYQPLAKKDPAIHTLLLGAINTQAEYVIESPYCNAFQPPPISDLPVSSNGQDDNVHP
AN7307.1  	QLANYQTLANRDRRLYNLIQGAINTQAEFVIQSPYCNAFQPPPPSNIPPEAHNQDDQVHP
          	**  ** **.::  :  *: ******:*::*:*********** :.:*   :.*** ***

MG04128.1 	NYDPNFVFECKYELDSLAHFLAIGNDFFEETGSTEFVNSRWLLALDTLLIVLEQQAMPTF
NCU09737.1	AFDPSSVFECKYELDSLAHFLALANAFYAHTKSTAFLNTRWYKALDSVFDVLEAQSKSTF
FG04255.1 	AYEPRAVFECKYELDSLAHFLALGNDFYDHTGSTDFVNKRWLRAVDTVLNVLTEQSKSTF
AN7307.1  	AYEPSVVFECKYELDSLANFLALTADFHENTGSTDFLTSRWYTALDTLLAVLDAQSQPTF
          	 ::*  ************:***:   *. .* ** *:..**  *:*::: **  *: .**

MG04128.1 	NSKTGSFRLNQYTFQRRTTLGTETLNLQGVGNPLGNGTGLVRSAFRPSDDASILGFFIPA
NCU09737.1	DPETGSYRRNQYTFQRRTDTGTETLNLGGIGNPLNNGTGLIRSAFRPSDDATIFGFFIPA
FG04255.1 	DPKTGEFQRNEYTFSRRTDTGTETLNLRGVGNPLNWGTGLVRSAFRPSDDATILGFFIPA
AN7307.1  	N-TEGQFVTNQYTFQRTTTLGTETLSLAGVGNPLNSGTGLIRSAFRPSDDATIMGFFIPA
          	:   *.:  *:***.* *  *****.* *:****. ****:**********:*:******

MG04128.1 	NAMMAVELGRAAKALKAA-DMADDTLTATVERWSKTITEAVWEHGVVKHKTHGDVFAYEV
NCU09737.1	NAQMATELKRTAALLRTTGNKKDVTRAETAEAWSKTITEGIWEHGVVHHRKYGDVFAYEV
FG04255.1 	NAQMSVELKRASKMLAAA---GKTTLSQKLESWSTKLREGVMEHGVIKHKKYGDVFAYEV
AN7307.1  	NAQMAVQLNKTAAMLRTAG--GHDDLANNLQDRSTRLRRAIQENAIVNHPKFGDVYAFEV
          	** *:.:* :::  * ::.   .   : . :  *. : ..: *:.:::* ..***:*:**

MG04128.1 	DGYGSAILMDDANYPSLLALPVMGFVSKEDKIYQNTRKMLLSKQSNPYYLEGRDFRGIGG
NCU09737.1	DGYGSALMMDDANYPSLLALPLMGFVSVGDTTYQNTRKMLLEKNGNPYYLEGKEFMGIGG
FG04255.1 	DGYGSSILMDDANYPSLLALPLMGFCEASDPIYQNTRKMLLEKSGNPYYLEGKGFKGIGG
AN7307.1  	DGYGSHVFMDDANVPSLLSLPVLGYLDKDDPVYQNTRKMILSKDGNPYYLTGSAFHGIGG
          	***** ::***** ****:**::*: .  *  *******:*.*..***** *  * ****

MG04128.1 	1PHI~GLQNAWPMSLLMQAQTTDDDEEIQGCLQLVLKSALLGLVHESVDVNQITSYTRSW
NCU09737.1	~PHI~GLQNAWPMSLLVQAQTSDDDDEIMKCLNLVLNTSKLGLIHESIDVNWSHSYTRKN
FG04255.1 	1PHI~GLRNAWPMSLLVQARTSDNDEEIKECIDLVLNSAKLGLVHESIDVEHISQYTRSW
AN7307.1  	~PHI1GLENAWPMSLLIQAQTSDSDIEIRECLNLVKNSSLLGLVHESINVNNIVEYTRPW
          	 *** **.********:**:*:*.* **  *::** ::: ***:***::*:   .***  

MG04128.1 	FAY1-----------LAKRKPYLIFKD-SEPYVI
NCU09737.1	SSS~-----------LGQDGRETWTKL-T-----
FG04255.1 	FAW~ANGVFASTILDLAKRKPHLIFGEGATAYEI
AN7307.1  	FAW~ANSVFAQTVLKIAAERPAIVFGEGAEPYIP
          	 :  :..  :.:  .:.          .: .
```
